# Measuring optic nerve sheath diameter using ultrasonography in patients with idiopathic intracranial hypertension

**DOI:** 10.1590/0004-282X-ANP-2021-0136

**Published:** 2022-05-20

**Authors:** Kenan Dağdelen, Merve Ekici

**Affiliations:** 1 Beytepe Murat Erdi Eker State Hospital, Department of Ophthalmology, Çankaya, Ankara, Turkey.; 2 Beytepe Murat Erdi Eker State Hospital, Department of Neurology, Çankaya, Ankara, Turkey.

**Keywords:** Pseudotumor Cerebri, Ultrasonography, Optic Nerve, Pseudotumor Cerebral, Ultrassonografia, Nervo Óptico

## Abstract

**Background:**

Idiopathic intracranial hypertension (IIH) is primarily a disorder of obese young women characterized by symptoms associated with raised intracranial pressure in the absence of a space-occupying lesion.

**Objective:**

To compare the mean optic nerve sheath diameter (ONSD) measured using ultrasonography (USG) in patients with idiopathic intracranial hypertension (IIH) and normal healthy individuals.

**Methods:**

A prospective study. Ninety-seven participants aged 18-80 years were divided into two groups as patients with IIH (n=47) and the control group (n=50). The ONSD was measured using ultrasound with a 10-MHz probe. ONSD was measured 3 mm behind the optic disc. Receiver operating characteristic (ROC) curve analysis was performed to determine patients with IIH using ONSD.

**Results:**

Body mass index was higher in the IIH group compared with the control group (p=0.001). The mean ONSD was statistically significantly thicker in the IIH group (6.4 mm) than in the control group (4.90 mm). The cut-off value of ONSD in patients with IIH was measured as 5.70 mm. There was a significant negative correlation between ONSD and age (r:-0.416 and p<0.001). There was a positive correlation between BMI and ONSD (r: 0.437 and p<0.001).

**Conclusions:**

Ultrasound can be a reliable, non-invasive and rapid tool to measure ONSD in monitoring patients with IIH. After the first diagnosis of IIH, based on neuroimaging and measuring intracranial pressure using invasive methods, ONSD can be used in treatment and follow-up.

## Resumo

**Antecedentes:**

A hipertensão intracraniana idiopática (HII) é primariamente um distúrbio de mulheres jovens obesas caracterizado por sintomas e sinais associados à pressão intracraniana elevada na ausência de uma lesão ocupante de espaço.

**Objetivo:**

Comparar o diâmetro médio da bainha do nervo óptico (ONSD) medido por ultrassonografia (USG) em pacientes com hipertensão intracraniana idiopática (HII) e indivíduos normais e saudáveis.

**Métodos:**

Estudo prospectivo. Noventa e sete participantes com idade entre 18-80 anos foram divididos em dois grupos: pacientes com HII (n=47) e o grupo controle (n=50). O ONSD foi medido por ultrassonografia com uma sonda de 10 MHz. O ONSD foi medido 3 mm atrás do disco óptico. A análise da curva ROC foi realizada para determinar pacientes com HII usando-se o ONSD.

**Resultados:**

O índice de massa corporal foi maior no grupo HII comparado ao grupo controle (p=0,001). O ONSD médio foi estatística e significativamente mais espesso no grupo HII (6,4 mm) do que no grupo controle (4,90 mm). O valor de corte do ONSD em pacientes com HII foi medido em 5,70 mm. Houve correlação negativa significativa entre ONSD e idade (r:-0,416 ep<0,001). Houve correlação positiva entre IMC e ONSD (r: 0,437 ep<0,001).

**Conclusões:**

A ultrassonografia pode ser uma ferramenta confiável, não invasiva e rápida para medir o ONSD no monitoramento de pacientes com HII. Após o primeiro diagnóstico de HII, com base em neuroimagem e na medida da pressão intracraniana por métodos invasivos, o ONSD pode ser utilizado no tratamento e acompanhamento.

## INTRODUCTION

Idiopathic intracranial hypertension (IIH) is quite common in routine neuro-ophthalmology clinics. The diagnostic criteria for IIH were first introduced by Walter Dandy in 1985^1^. Clinically, it progresses with headaches and loss of vision-visual field in young-middle-aged women^2^.

In evaluating patients with IIH, lumbar puncture is generally preferred invasively, and magnetic resonance (MRI) and computed tomography (CT) noninvasively. Although invasive techniques are accurate and highly sensitive, they can cause adverse effects such as hemorrhage and infection that need to be managed^3^. However, CT and MRI are time-consuming, costly, and require patient transport. Therefore, evaluation of optic nerve sheath diameter (ONSD) using ultrasonography (USG), which provides low-cost and fast bedside examination, is a better option, especially in cases where patient transport is difficult, such as in the intensive care unit (ICU)^4^ ^-^ ^7^. The technique is cheap and effective, and examinations take approximately 5 minutes at the bedside^8^. ONSD has been measured as retrobulbar at a distance of 3 mm in most studies^9^ ^,^ ^10^. The optic nerve is surrounded by a dural sheath as part of the central nervous system. There is a small subarachnoid space of 0.1-0.2 mm between the dural sheath and white matter and communicating with the subarachnoid space surrounding the brain. When intracranial pressure (ICP) increases, the dural sheath expands and changes in the diameter of the sheath can be demonstrated using transocular USG^5^. 

This study was conducted to review the effectiveness of USG in the evaluation of patients with IIH and to compare the findings with other studies in the literature.

## METHODS

**Figure 1.**
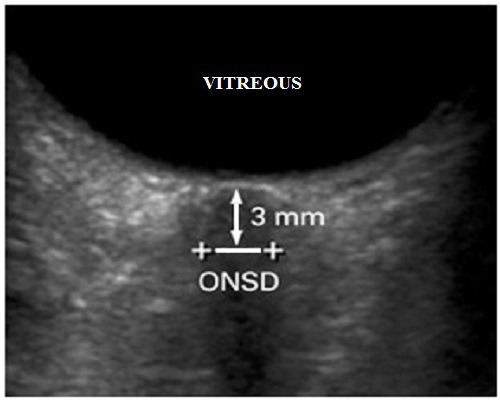
Transverse length of the optic nerve sheath, starting 3 mm behind the optic nerve head.

This study was conducted as a case-control study in Ankara Provincial Health Directorate Beytepe Murat Erdi Eker State Hospital department of ophthalmology and neurology, Ankara, Turkey in the first quarter of 2021. The study protocol was approved by the ethics committee (registration number is E1/1541/2021). Fifty healthy volunteers and 47 patients with IIH were included in the study. The inclusion criteria for the study were as follows: age 18-80 years, diagnosed with IIH for a maximum period of 12 months, no additional disease, no drug history, no active or previous intraocular and orbital infections, no ophthalmologic disease other than refractive error, no history of eye-orbital-cranial surgery, less than -5.00 D (Diopter) and +3.00 D refractive error, no history of eye or head injury, no history of radiotherapy to the head and orbital region. Informed consent was obtained from all participants. The diagnosis of IIH was made by a neurologist according to the Dandy criteria. The patients underwent detailed neurologic examinations, ICP measurements, and lumbar puncture by a neurologist. Patients in the IIH group were also subjected to examinations by an ophthalmologist to exclude other causes of optic disc edema and diagnose papilledema. Participants were divided into two groups as patients with IIH and healthy volunteers. Measurements were taken in the supine position using a 10-MHz probe speed real-time ultrasound device (ultrasound scanner model E-Z Scan 5500+ by Sonomed Inc. NY) from the participants in both groups, with the probe placed in the superolateral of the globe with the upper eyelid closed. Only one eye of all participants was evaluated. The optic nerve head was visualized as a linear hypoechoic structure. ONSD was measured three times by the same investigator and the mean value was calculated. ONSD was measured 3 mm behind the optic nerve head (optic disk) as the transverse length of the optic nerve sheath (Figure 1). 

### Statistical analysis

The data were recorded on a spreadsheet and analyses were performed after transfer to the SPSS version 25 software. Frequency and percentage values were used to describe categorical data, and Chi-square tests were used for comparison. The compatibility of continuous data to normal distribution was tested using the Shapiro-Wilk test. The continuous data did not conform to normal distribution and parametric assumptions were not met; thus these variables were described with median and 25-75% interquartile range (IQR) values. The Mann-Whitney U test was used to compare quantitative variables. Spearman’s correlation coefficient was used for the comparison of continuous variables. Receiver operating characteristic (ROC) curve analysis was performed to determine patients with IIH using ONSD. Any p-value of <0.05 was accepted as demonstrating significance. 

Power Analysis was performed through the PASS 11 program. Using the ONSD values of the patient and control groups in the study by Rehman et al.^2^, it was concluded that each group should consist of at least 35 people with an 80% power level and 0.05 alpha errors.

## RESULTS

The mean disease duration of patients with IIH was 3.85 ± 2.67 months. Ninety-seven eyes of 97 patients included in the study were examined. There was no significant difference between the two groups in terms of right and left eye distributions (p=0.917). In the IIH group, 36 (76.6%) people were female and 11 (23.4%) were male, and in the control group, 34 (68.0%) were female and 16 (32.0%) were male (Table 1).

**Table 1.** Distribution of the two groups by sex and eye care included.

		Group		P value
		IIH n (%)	Control n (%)	
Eyes	Right	23 (48.9%)	25 (50.0%)	0.917
	Left	24 (51.1%)	25 (50.0%)	
Sex	Male	11 (23.4%)	16 (32.0%)	0.345
	Female	36 (76.6%)	34 (68.0%)	

IIH: idiopathic intracranial hypertension.

The mean age was 28 (range, 26-32) years in the IIH group, and 38 (range, 32-45) years in the control group; the IIH group was significantly younger (p=0.001). Body mass index (BMI) and blood pressure (diastolic) were higher in the IIH group compared with the control group (p=0.001 and p=0.039, respectively). The mean ONSD was statistically significantly thicker in the IIH group (6.4 mm) compared with the control group (4.90 mm) (p=0.001). There was no significant difference between the groups in terms of spherical equivalent (diopter), glycated hemoglobin (HbA1C) (mMol0/L), and blood pressure (systolic) values. The demographic data and examination findings of the two groups are given in Table 2. In the IHH group, 57.4% of the cases were receiving acetazolamide treatment.

**Table 2.** Demographics and optic nerve sheath diameter values in controls and patients with Idiopathic intracranial hypertension.

	Group		P-value
	IIH n (%)	Control n (%)	
Age	28 (26-32)	38 (32-45)	0.001*
Spherical equivalent (diopter)	-1.00 (-2.00-1.00)	-1.00 (-2.00-1.00)	0.663
Body mass index	34.81±5.33	28.58±3.91	0.001*
Blood pressure (systolic)	120 (110-130)	117.5 (105-120)	0.097
Blood pressure (diastolic)	80 (70-85)	72.5 (70-80)	0.039
HbA1C (mMol/L)	4 (3.5-4)	3.5 (3.5-4)	0.279
Optic nerve sheath diameter	6.4 (6-6.7)	4.9 (4.6-5.2)	0.001*

*: statistically significant, p-value <0.05; IIH: idiopathic intracranial hypertension.

Table 3 shows the correlation of the ONSD and demographic data. A high level of negative correlation was observed between ONSD and age (r: -0.416 and p<0.001). A high level of positive correlation was observed between BMI and ONSD (r: 0.437 and p<0.001). Moderate positive correlation was observed between HbA1C (mMol/L) and ONSD (r: 0.227 and p=0.025). There was no significant correlation between ONSD and disease duration, blood pressure, and spherical equivalent. The mean cerebrospinal fluid (CSF) pressure was 269.72 ± 66.29 mmH_2_O in the IHH group. A high level of positive correlation was determined between CSF pressure and ONSD (r: 0.740 and p<0.001).

**Table 3.** Correlation of optic nerve sheath diameter and demographics.

		Optic Nerve Sheath Diameter (mm )		
		Total	IIH	Controls
Age	r	-0.416*	0.039	0.118
	p	<0.001*	0.793	0.416
Disease duration (months)	r	-0.160	-0.160	-
	p	0.282	0.282	-
Spherical equivalent (Diopter)	r	-0.006	-0.179	0.272
	p	0.953	0.228	0.056
Body mass index	r	0.437*	0.051	-0.263
	p	<0.001*	0.733	0.065
Blood pressure (systolic)	r	0.120	0.020	-0.121
	p	0.241	0.896	0.404
Blood pressure (diastolic)	r	0.095	-0.203	-0.144
	p	0.356	0.171	0.318
HbA1C (mMol/L)	r	0.227*	0.353	0.178
	p	0.025*	0.015	0.217

*: statistically significant p-value of <0.05; HbA1C: Hemoglobin A1c.

**Figure 2.**
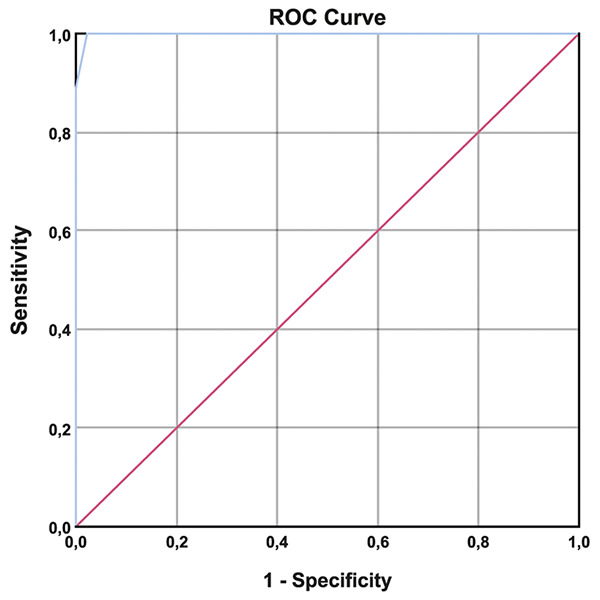
Receiver operating characteristic (ROC) analysis.

According to the ROC analysis, the cut-off points of 5.70 mm, showed 100% sensitivity and 98% specificity (AUC: 0.999 [0.996-1.000]) (Figure 2).

## DISCUSSION

In our study, the efficiency of USG in the evaluation of patients with IIH and the measured mean ONSD value and cut-off value was compared with other studies in the literature. IIH is seen in young women. ONSD was highly correlated with age (r: -0.416, p<0.001) and BMI (r: 0.437, p<0.001). The cut-off value of ONSD in patients with IIH was determined as 5.70 mm.

ONSD measurement using USG is a simple, fast, non-invasive, and reliable method. Although lumbar puncture is the gold standard for measuring ICP, studies have reported that ONSD has a positive correlation with ICP^11^ ^-^ ^13^. ONSD values are measured at a fixed distance, and measurements taken as retrobulbar at a distance of 3 mm are considered reliable in the literature^2^ ^,^ ^9^ ^,^ ^14^ ^,^ ^15^. In our study, the average ONSD values were calculated using B-mode USG from a retrobulbar 3 mm distance. However, as with all USG evaluations, it requires training. It has intra and interobserver variance, but these variations are small. In recent studies, the mean intraobserver variance was found as ± 0.1-0.2 mm, and the mean interobserver variance was ± 0.2-0.3 mm^9^ ^,^ ^10^. ONSD can also be measured using MRI, but the patient must be transported for MRI. Bedside evaluation of patients provides a great advantage, especially in ICPs and emergency departments. Studies comparing USG and MRI on this subject are also available in the literature. In a study comparing ONSD values measured using USG and MRI, it was concluded that ONSD values taken from a retrobulbar 3 mm distance showed a high level of correlation between the two methods^16^. Chen et al. showed ultrasonographic measurements of ONSD can dynamically and sensitively evaluate real-time ICP. In their study, ONSD measurements were performed approximately 5 min prior to and after a lumbar puncture. They found significant correlations between ONSD and ICP before lumbar punctures and between the median change in ONSD and the change in ICP^17^. Padayachy et al. reported in child patients, the ONSD measurement with the best diagnostic accuracy for detecting an ICP ≥ 20 mmHg over the entire patient cohort was 5.5 mm, sensitivity 93.2 %, specificity 74 % and odds ratio of 39.3^18^. Tekin Orkun et al. also indicated the mean cerebrospinal fluid opening pressure (37.75 ± 12.64 cm H_2_O) and the mean ONSD (5.94 ± 0.46 mm) were correlated in small sample size of pediatric IIH patients^19^. In contrast to these studies, Lochner et al. reported no correlation was demonstrated between ONSD and cerebrospinal fluid opening pressure in adult IHH patients. They also found no differences in optic nerve diameter values between patients and control groups^20^.

In our study, in accordance with the literature, ONSD values were significantly higher in the IIH group than in the control group^2^ ^,^ ^9^ ^,^ ^10^. As frequently reported in the literature, IIH is mostly seen in young women with obesity^1^ ^,^ ^2^. Zheng et al. found a correlation between ONSD and BMI using high-resolution MRI. Their result indicates that the effects of BMI should be considered along with the ONSD during ICP monitoring. Meanwhile, the correlation index between ONSD and BMI was better than the ONSD in predicting IIH and could be used to obtain a more precise estimation of ICP^21^. As a different point of view, Lochner at al. claims to monitor the efficacy of diet and pharmacological treatment in IIH patients^22^. In our study, it was observed that the IIH group consisted mostly of young female patients with higher BMI values. In addition, a high level of correlation was observed between ONSD values and age (r: -0.416, p<0.001) and BMI (r: 0.437, p<0.001).

There is no single cut-off value for ONSD. This should be considered as a clear indication of abnormal or elevated ICP. In our study, the mean ONSD value was 6.40 mm in the IIH group and 4.90 mm in the control group. The best ONSD cut-off value indicating increased ICP was determined as 5.70 mm (100% sensitivity and 98% specificity) with an area under the curve (AUC) of 0.999. Shrestha et al. concluded that 95% of normal individuals in Nepal had an average ONSD value of 4.41 mm^23^. Dubourg et al., in their meta-analysis, concluded that ONSD had a cut-off value of 5.10 mm^24^. In a study conducted in China, Wang et al. reported that the mean ONSD value was 4.33 ± 0.38 mm in normal individuals and 6.61 ± 0.39 mm in patients with IIH^25^. Kishk et al. measured the ONSD cut-off value as 6.05 mm (73.2% sensitivity and 91.4% specificity) with an AUC of 0.850^14^. Fernando et al. showed in their study in patients with increased intracranial pressure for various reasons, the pooled AUC curve for ONSD sonography was 0.94 (0.91 to 0.96)^26^. Li et al. reported in their study AUC analysis showed the ONSD of 5.6 mm was the best cutoff value with a sensitivity of 86% and a specificity of 71% for identifying high ICP^27^. Del Saz-Saucedo et al. found that the best cut-off point for detecting raised ICP was 6.3 mms, with a sensitivity, specificity and positive likelihood ratio of 94.7%, 90.9% and 10.4, respectively. After a therapeutic lumbar puncture an 87% of cases had a partial reduction of ONSD values^28^. According to the ROC analysis of our study, the cut-off point of 5.70 mm, showed 100% sensitivity and 98% specificity (AUC: 0.999 [0.996-1.000]). The sensitivity and specificity rates are the highest rates when compared to the other studies. Despite considerable debate on the normal and abnormal cut-off value of ONSD in different populations, this ultrasonographic measurement easy and noninvasive research method cannot be underestimated.

The limitation of our study is that when ONSD values are measured using USG, patients' ICPs are not measured, and they cannot be compared with ONSD values. Secondly, the distribution of age was different between the IHH and control groups. Some studies showed a correlation between age and ONSD^29^, while others did not^30^. In our study, age was negatively correlated with ONSD. The heterogeneous distribution of age in the groups may also have affected the ONSD value. This was not taken into account when interpreting our results.

In conclusion, USG is a reliable, cheap, non-invasive and fast tool for measuring ONSD in the monitoring of patients with IIH. Although many studies have been conducted on the ONSD cut-off value as measured using USG, there is no consensus among authors yet. Our study made significant contributions to the literature in terms of determining ONSD cut-off values and demonstrating the diagnostic value of ONSD in patients with IIH. The values of the optic nerve sheath can replace cerebrospinal fluid pressure in the diagnosis or follow-up of these patients. Studies on this subject should be conducted on larger data sets and with a longer follow-up period. Considering the results of our study and comprehensive future studies, ONSD value may be considered as a preferable option in the diagnosis of IIH patients.
